# Digital exclusion faced by people living with chronic respiratory disease: Challenges, implications and solutions

**DOI:** 10.1177/14799731261441307

**Published:** 2026-04-03

**Authors:** Grant Trewartha, Lotte Janssens, Samantha Harrison

**Affiliations:** 1 School of Health and Life Sciences, 5462 Teesside University, Middlesbrough, UK; 2 REVAL Rehabilitation Research Center, UHasselt, Hasselt, Belgium

**Keywords:** digital health technologies, barriers, digital divide, COPD

## Abstract

The introduction of digital health technologies (DHTs) to support the diagnosis, assessment, and management of chronic respiratory diseases (CRD) has great potential to democratise access to healthcare, but will suffer if these new technologies cannot be fully utilised by the patients most at need, particularly those from underserved communities. This narrative review addresses the challenges and potential solutions for reducing digital exclusion for people living with CRD. Although sparse, the available evidence suggests that digital exclusion leads to poorer health outcomes in people living with CRD. The barriers that lead to digital exclusion intersect at many different levels and include socioeconomic, demographic, geographical, digital literacy, design/accessibility and psychosocial factors. Solutions to mitigate digital exclusion in people with CRD need to operate at multiple scales with cross-sectoral collaboration, and range from ensuring access to digital tools via large national mobile/broadband infrastructure developments to ensuring DHTs for patient use are designed inclusively and frontline healthcare staff are trained to help patients engage with the tools. Currently, there is a real risk that deploying digital health interventions for CRD care may widen the digital divide and deepen health inequities. To deliver on the digital health promise, all relevant stakeholders need to be focussed on ensuring that the presence of digital exclusion is well monitored and underserved communities such as CRDs are not systematically excluded from implementation and evaluation efforts.

## 1. Introduction and aims

Chronic respiratory diseases (CRD) are a leading and increasing^1^ cause of disability and mortality worldwide, with prevalence varying considerably by region, age, sex and gender, ethnicity, and socioeconomic status. Individuals living with CRD face various challenges, which include physical limitations (e.g. reduced mobility, breathlessness, fatigue), fluctuating symptoms and exacerbations, complex treatment regimes, social and environmental barriers (e.g. poor housing conditions), limited access to specialist care, comorbidities, limited health literacy, and mental health issues (e.g. anxiety, depression, feelings of isolation). Solutions to improve health outcomes for people with CRD include early diagnosis, close monitoring, appropriate therapy, and accessible health care.^1,2^ Conceivably, digital health technologies (DHTs), targeted for clinicians and patients, can provide a route to each of these solutions, but only if they can be fully utilised.^3^ Policy makers have also recognised the promise of digital transformation leading to improved public health, with better self-management for people with long-term conditions^3–5^ to reduce demand for healthcare services^6^ and free up resources for other areas.^7,8^ However, while the potential of DHTs to support long-term conditions such as CRD seems obvious, actual evidence of clinical effectiveness is scarce.

This lack of adoption might be explained by an important paradox in the digital health revolution, namely on one side the great potential for digital health innovations to transform care delivery to underserved groups (e.g. rural areas, older adults, ethnic minorities, people with disabilities); on the other hand, these same populations are often the most vulnerable to digital exclusion, largely due to their sociodemographic characteristics.^9–11^ Without due care and governance, the introduction of DHTs risks exacerbating health inequalities^12–14^ and generating a digital divide.

Therefore, the guiding research question that this narrative review addresses is **“What are the challenges and potential solutions for reducing digital exclusion for people living with CRD?”**.

The objectives of this narrative review are to:

1. Assess the impact of digital exclusion on disease outcomes and healthcare access for people with CRD;
2. Examine barriers to digital health access in people with CRD;
3. Provide practical recommendations on how to mitigate digital exclusion for people with CRD.

To prioritise a broad applicability, we have taken a ‘disease-agnostic’ approach by considering CRDs in general as the underlying issues are shared across multiple CRD conditions. The issues covered in this review are most relevant to high-income countries, and therefore we leave specific consideration of digital exclusion in low- and middle-income countries for a separate review. Lastly, whilst not excluding DHTs developed for diagnosis and assessment purposes, for a focus on digital exclusion most of the considerations relate to DHTs introduced for monitoring and management of CRD.

Initial literature scoping was conducted in August 2025, covering date ranges 2010-present and across CINAHL Ultimate, Embase, Web of Science and Medline databases. An example of the search terms used (for CINAHL) is given here: ((respiratory or pulmonary or COPD or asthma or lung disease or CRD) and (digital health or digital medicine or electronic health or ehealth or mhealth) and (digital exclusion or digital divide or digital poverty or digital inequality or digital equ*)). Additional literature was accessed via the reference lists of relevant articles.

## 2. Digital health technologies for CRD care – main concepts

DHTs are defined as any digital product or emerging technology used to enhance health and wellbeing or to improve health or social service systems.^15^ The use of digital health has greatly expanded^16^ and is expected to continue to grow at a rapid rate^17^ due to technological advances, for example in AI tools, and the spread of mobile phones and other digital tools.^18^ Smartphone ownership is almost universal amongst the working-age population in Northern Europe^19^ although this usage drops significantly for older adults and in other regions of the world.^20^ The implementation of DHTs was greatly accelerated due to and during the COVID-19 pandemic when face-to-face healthcare was restricted.^21^ Within the UK for example, NHS App registrations increased from 2 million people in 2021 to 30 million in 2023.^22^

DHTs have been employed broadly in the management of CRD, including telemedicine with virtual consultations,^23^ remote patient monitoring such as home spirometry and pulse oximetry,^24^ digital pulmonary rehabilitation (PR) programmes,^25^ and mHealth apps for symptom tracking and medication adherence.^26,27^ Pimenta et al. (2023) provided a comprehensive summary of how to tackle ‘pain points’ in PR by using digitally-supported delivery models of PR, for example, improvements to: access (reducing travel barriers through remote delivery), uptake (aligning with patient preferences), and completion (minimising attendance burden and supporting continuation of PR during exacerbations).^28^

Benefits of DHTs for CRD care have been shown across a number of health outcomes,^29–32^ including reduced emergency department attendance,^33^ reduced hospitalisation rates,^34^ decreased frequency of exacerbations,^35^ improved adherence to medication and physical activity,^27,35–37^ better self-management of COPD in rural areas,^38^ and promotion of health-seeking behaviours in underserved patients who might typically avoid consultations and ignore warning signs.^39^ Studies have concluded that both healthcare professionals^27^ and patients^40^ perceive DHTs to facilitate autonomy and improve the quality of self-management of people with CRD. Cooper et al. (2022) conducted a real-world feasibility study for the “myCOPD” digital care management tool in a rural setting and found, counter to some other data, that age, income and geographical location did not represent significant barriers to patients using myCOPD and presented these findings as an indication that DHTs could be deployed as part of routine care provision without increasing health inequalities.^41^

Nevertheless, there are obstacles to proceeding at pace and scale with a “digital first” approach for CRD care. The digital divide refers to structural and social disparities in both access to and effective use of digital technologies, such as computers, smartphones, and reliable internet connectivity, encompassing not only physical availability but also the skills required to use these tools.^11,42^ According to Age UK, one in five older adults have no or very little access to the internet and internet usage among older adult groups is decreasing.^43^ As healthcare becomes increasingly technology-driven, the digital divide risks deepening existing disparities^44^ and undermining the success of digital health implementations.^45^

Digital exclusion is the lived experience or consequences of the digital divide. Whereby individuals or groups are unable to participate fully in digital society, including access to essential services such as healthcare.^11,42,46,47^ Certain groups and individuals are at higher risk of digital exclusion, such as minority ethnic groups, socially disadvantaged communities, older people, and people with disabilities.^48,49^ The key issues typically relate to lower affordability, access, and ability (digital skills, self-efficacy, barriers related to disability). Digital exclusion occurs because of a mix of factors including age, socioeconomic status, disability, geography, educational attainment, literacy and language, and housing situation^50^ with many of these contributing factors known to disproportionally impact people living with CRD.^51,52^ Digital exclusion is not a static concept and can vary due to changing circumstances and life stage.^53^ Large institutions increasingly acknowledge the negative consequences of digital exclusion and have taken steps to reduce resulting disparities.^54,55^ However, the full extent of the barriers and the impact of digital exclusion on people with CRD remains unknown.

## 3. The impact of digital exclusion on CRD health outcomes

Currently, much of the evidence for the impact of digital exclusion on health outcomes relies on a broader consideration of people living with chronic conditions, rather than specifically people living with CRD, although the evidence linking digital exclusion to poorer outcomes for individuals with CRD is in-part established through studies that have demonstrated the positive impact of inclusion (see Section 2). Honeyman et al. (2020) explain that digital exclusion can negatively affect population health outcomes through two pathways: direct routes, where patients are unable to benefit from digitally delivered health services such as telehealth consultations or remote spirometry, and indirect routes, where access to broader determinants of respiratory health, such as housing support or employment opportunities, becomes dependent on digital connectivity.^56^ Evidence for the presence of digital exclusion and a widening of the digital divide for groups of older adults living with socially-patterned chronic diseases has been shown^14,57,58^ but is not extensive, one issue being that digital exclusion is often not specifically measured as part of implementation efforts.

Digital exclusion is multidimensional, beyond mere access, and includes affordability, adequacy, availability, and acceptability considerations. These factors can affect patients’ ability to engage with digital health services,^47,59,60^ keeping them from benefits such as reduced travel and travel planning, timely care interventions, and continuous monitoring. Patients who are unable, unwilling or lack confidence to engage digitally are also less likely (and have less satisfaction^61^) to participate in home-based monitoring, online PR, and alert/feedback services,^25,28^ all crucial for early intervention and acute exacerbation prevention. To compound the issue, the viewpoints of patients who don’t use technology are rarely captured or considered.^7^ Therefore, patients who are digitally excluded are less likely to benefit, potentially resulting in higher healthcare utilisation and worse health status. Before proposing some solutions to reduce the impact of digital exclusion within CRD management, it is firstly important to take a closer look at each of the contributing factors.

## 4. Barriers to digital health access in people with CRD

The prevailing consensus suggests that unequal access to DHTs exacerbates health disparities among those CRD groups with the greatest unmet healthcare needs.^14,62,63^ In addition to the more person-centric barriers which will be considered fully in this section, it has been recommended that research which aims to understand digital inequalities and exclusion should also bear in mind the broader context, including the policy environment, culture and societal values.^14^

### 4.1. Socioeconomic barriers

Lower income and social disadvantage are strongly associated with lack of access to the internet and digital devices. Respiratory conditions, such as COPD, are more prevalent in these groups,^64^ which increases their risk for digital exclusion. The cost of devices, data plans, and broadband is viewed as a significant obstacle to engaging with DHTs,^65,66^ and digital use may be reducing in some population groups due to affordability concerns.^46^ In UK households from the lowest socioeconomic backgrounds, 21% do not use the internet at home, and 38% are classified as ‘narrow’ users who rarely participate in online activities such as banking, finding information, and searching for public services.^67^ There appears to be an inverse relationship between socioeconomic status and take-up of digital health interventions. An analysis of NHS app activations across 6356 primary care providers in England found a significant association between increased population deprivation and reduced digital utilisation and concluded that technology is driving widening of healthcare inequalities.^68^

In addition to the direct influence of socioeconomic status on digital health access through financial barriers, digital literacy plays an important mediating and moderating role.^12,69^ A NICE evaluation of digital self-management technologies in people with COPD, who tend to be older and from lower socioeconomic backgrounds, concluded that this patient group might be less comfortable and possess fewer skills for using digital technologies in addition to the increased likelihood of not having the required devices.^7^ Much of the evidence linking socioeconomic status to digital exclusion in CRD management comes from high-income countries; this disparity is likely even greater in low- and middle-income settings.^28^

### 4.2. Demographic & health-related barriers

Older age remains one of the most significant predictors of digital exclusion in general. In the UK, 31% of people >65 years do not use the internet at home, compared with just 4% for those aged 35–44 years.^46^ Older adults are less likely to have digital skills, confidence, and/or access to technology, and are using digital health interventions less frequently.^27,66,70^ They also more often report difficulty in using the devices required.^36^ COPD is a multimorbid condition and older adults with COPD will often have other health conditions (such as arthritis, heart disease, diabetes) or mobility limitations, making it harder to use digital technology or attend digital skills training. Visual or cognitive impairments are also common,^71^ and manual dexterity limitations pose additional challenges.

There are evident racial and ethnic disparities in the uptake and effectiveness of digital health interventions for chronic conditions including CRD. A systematic review of 34 qualitative studies found that ethnic minority status (a range of culturally and linguistically diverse populations) was consistently associated with barriers to digital health engagement, including low digital literacy, language barriers, cultural mismatches in design, and distrust in digital systems.^72^

Educational level/attainment is another demographic factor that can act as a barrier to uptake of digital health tools, and this factor may intersect with other demographics such as socioeconomic status. It has been suggested that individuals with (severe) asthma and COPD tend to have both poor educational attainment and lower socioeconomic status.^12^ This can challenge implementation of digital health tools and widen health disparities since the most educated make the best use of new information and adopt newer technologies first.^54^

### 4.3. Technological barriers & digital health literacy

Digital health literacy can be defined as a person’s ability to search, acquire, comprehend and appraise health information from digital technologies with the goal of improving their quality of life.^28^ Low digital health literacy has significant consequences. It can deepen health inequities, as patients who lack the skills, awareness, or language to use digital tools often disengage.^11,54^ This limits opportunities for health improvement. For this reason, digital health literacy is increasingly recognised as a super social determinant of health, given its influence on broader social determinants.^10^

Healthcare professionals often express concern about patients’ low digital health literacy^28^ and anticipate that adding digital interventions to already complex treatment plans could increase the risk of disengagement.^73^ Whether the low digital health literacy of patients is real or only perceived, it may well lead to decreased adoption of DHTs since the health professionals will act as gatekeepers to the adoption of DHTs. Conversely, enhancing digital health literacy can improve acceptance and use of DHTs among key patient groups.^74^ For example, older adults with chronic conditions who actively engaged in sharing health resources via DHTs reported greater confidence in using these tools and strengthened social connections.^75^

From a technological know-how and skills training perspective, adequate training in DHTs is essential for both patients and health care providers to ensure positive health-seeking behaviours and to encourage implementation. Barriers to DHTs can come from healthcare professionals due to their own limited digital health literacy, a sense of hyperconnectivity and concerns about increased workload,^39,40^ especially if technologies are used as an adjunct to, rather than a replacement of, usual care.^7^ There may also be a more fundamental scepticism in the approach emanating from health professionals, with doubts about the reliability and efficacy of DHTs.^27,40,76^

### 4.4. Geographical barriers in digital (health) access

DHTs have the potential to benefit geographical accessibility to health by extending reach to remote and underserved areas. Unfortunately, direct evidence for these benefits is limited.^77^ Multiple factors such as cost of transport, lower health literacy,^78^ lack of access to critical services such as PR^79^ and poorer lifestyle behaviours already feed into an urban-rural divide that produces higher burden of CRD in remote communities.^80^ Adding difficulties in digital health access for these rural communities^81^ leads to disparity in the extent to which DHT interventions are deployed for people with CRD in more remote locations (rural and coastal).^22^ There is therefore an inclusivity challenge if services become “digital by default” unless alternatives are maintained.^82^

### 4.5. Design and accessibility barriers

There is also the problem of applicability/personalisation for deployed DHTs, many technologies are a one-size-fits-all form and are not usually tailored to the specific diverse needs or characteristics of the patient or patient groups. For example, people with COPD often have multimorbidity^83^ and require integrated management tools that address cardiovascular or metabolic conditions alongside respiratory care, yet most apps have a narrow focus. Similarly, paediatric asthma interventions benefit from gamified features and parental engagement,^84^ whereas adult asthma tools could assume self-management autonomy. Patients with low health literacy or limited digital skills, often older adults or those with lower education,^85^ struggle with complex interfaces, and ethnic or cultural differences in asthma triggers and language preferences are frequently overlooked.^86^ DHTs should also be customised depending on condition severity, individuals with advanced COPD may need oxygen therapy integration and real-time symptom alerts, while those with mild disease benefit more from exercise coaching and smoking cessation support. The lack of representation of minority groups in health datasets, which digital health apps may rely on for giving care recommendations, contributes to health data poverty, reinforcing the digital divide through biases embedded in these datasets.^54,87^

Digital health platforms that are not designed inclusively— lacking large fonts, accessible navigation, or language/audio assistance—pose further barriers to adoption. Poor compatibility with assistive technologies further excludes those with sensory or physical limitations. Even where DHTs have been designed with accessibility in mind, their applicability can be compromised due to information overload, complexity of content, complicated user interfaces, and lack of contextualisation.^54^ Smartphone usage is socially patterned,^88^ therefore digital health tools which are developed with restrictions on the devices/platforms (e.g. iOS/iPhone only) may disproportionally benefit more advantaged populations.

Challenges in user adoption often stem from insufficient involvement of key stakeholders, such as patients, clinicians, industry partners, and regulators, during the design phase.^28^ When user needs are assumed rather than addressed, interventions are less likely to be perceived as valuable, increasing the risk of disengagement. To mitigate these issues, user-centric approaches, including human-centred design, are increasingly employed as foundational frameworks for developing digital health interventions.^89,90^ As a positive example, Metting et al. (2023) collected the opinions of patients (>55 years) with asthma and COPD following use of a pharmacy-based web application. They concluded that it is feasible to develop websites for this patient group; however, developers must take the specific needs and limitations of the patients into account, particularly in relation to page layout and navigation, and these aspects can be best improved via co-creation with target users.^12^

### 4.6. Social and psychological barriers

This section will discuss a number of social and psychological factors that can contribute to digital exclusion in CRD populations, but it is important to note at the outset that the presence of digital exclusion per se can have an effect and perpetuate social exclusion and isolation in people with CRD, a situation which is known to be associated with negative outcomes and already a problem in elderly and underserved CRD populations.^20^ Lower engagement in DHTs is observed in certain ethnic groups and among people already experiencing social isolation.^91,92^

In terms of facilitators, pre-existing positive attitudes to technology of both patients and HCPs may be a facilitator for increased uptake of DHTs.^36,40,93^ Similarly, pre-existing positive experiences of digital health interventions in terms of comfort and convenience influences future patient uptake.^18^ Some patients have been reported to be reassured by the additional “surveillance” afforded by involvement in digital health interventions.^7^ Individuals with COPD have reported several potential benefits relating to self-management via digital tools, which suggests there is some appetite among patients to adopt digital-led approaches.^73^

On the flip side, in terms of barriers, lack of engagement in DHTs may be linked to community beliefs and practices around data privacy, with some cultures being more sensitive to personal health data being transferred. Other barriers to acceptability and uptake of new technologies include lack of interest,^94^ lack of motivation of patient or HCP,^27,65,66^ loss of human contact,^39,94^ negative attitudes to technology,^27,40,93^ and perceiving technology as difficult to learn.^40^ A scoping review concluded that the major barriers to patients adopting digital health interventions for COPD management include poor digital literacy, perceptions that care delivery is impersonal, and a fear of being “controlled” by telemonitoring data.^95^ These barriers were broadly similar to those identified in a qualitative study on patient perceptions, which highlighted lack of perceived usefulness of the DHT, limited digital health literacy, and illness perceptions as the primary obstacles.^96^

Healthcare professionals can limit uptake of DHTs. Barriers from the perspective of HCPs include lack of motivation, anticipation of increased workload with no compensation for the HCP,^39,40^ and fear of hyperconnectivity. Healthcare professionals often cite the lack of robust evidence demonstrating that digital health interventions improve patient outcomes or reduce costs and workload as a key barrier to their adoption.^73^

To summarise, digital exclusion among people with CRD is primarily driven by a blend of socioeconomic disadvantage, age, health limitations, low digital health literacy, limited support, poor technology design, and social factors. Tackling this requires strategies that address each of these root causes.

## 5. Potential solutions for mitigating digital exclusion for people living with CRD

Policy organisation such as the Good Things Foundation in the UK have been active in proposing guidance to mitigate the risk of digital exclusion in health systems more generally.^97^ Their priority domains for action include: Access to devices and data connectivity; Accessibility and ease of using technology; Skills and capability (among the public and healthcare professionals); Beliefs and trust (among the public and healthcare professionals); Leadership and partnerships.

### 5.1. Access to devices and data (government & policy makers)

Improving access to affordable digital tools seems like the most systemic and challenging domain on which to make progress by the healthcare community working in isolation, the solution lies mostly at governmental level. Improved access requires internet infrastructure in the form of improved broadband coverage, particularly roll-out to rural and underserved communities and/or governmental support for 5G rollout and public wi-fi initiatives. These are massive infrastructure decisions and projects that are fundamental pre-cursors for successful digital health interventions to be built on but go beyond the sphere of influence of the health sector in isolation. Fortunately (in some respects) issues of digital exclusion go beyond healthcare and have serious implications for the economy, education, finance sector and more. Because of its wide-ranging impact, the healthcare sector can continue to lobby for improved digital access for the benefit of public health, by “piggy backing” on infrastructure improvements that are being made for a myriad of reasons.

It may be possible for healthcare providers to take a more proactive role in improving access in some cases. For example, in certain private healthcare situations (insurers or health providers) or for very high-risk populations there is a strong business case for the healthcare provider to distribute subsidised devices (phones, activity trackers, etc) and mobile internet access to patients. It is to be anticipated that these solutions will not be available to all patients and at scale.

Government strategies to improve access to digital health tools need to be tailored to regional needs, but could include initiatives like digital dispensaries, partnering with telecommunication companies to release affordable mobile phones, maintenance of public libraries which have free internet access, device lending schemes, and digital health literacy training.

### 5.2. Accessibility and ease of use of DHTs (Developers & innovators)

Improving accessibility of DHTs is a factor much more under the control of the healthcare innovation community. It has been suggested that the digital divide can be reduced through careful design and implementation of DHTs into routine healthcare.^98^ Careful design would include ensuring inclusivity through aspects such as simple app layouts, ease of use, low literacy requirements, audio options, and multilingual support. User-centred design and personalisation of digital health apps based on patient and HCP needs identified via extensive Patient and Public Involvement and Engagement (PPIE) and co-creation activities may be key to improving patient engagement.^20^ Digital health providers also need to ensure interoperability with other key systems such as electronic health records and the scalability of apps, whilst evaluating the impact of different technologies on HCP workload and existing services.^99^

Digital health technologies should also be designed to address the specific needs of disadvantaged groups.^56^ Even with technical design directed to make tools broadly accessible, content creation also needs to consider other limitations which might impair the vision, hearing, speech, processing, or memory of users. Inclusive design approaches also need to be targeted to counter bias across economic and racial groups.^49^

The Digital Health Care Equity Framework (DHEF) developed by Johns Hopkins University offers a roadmap for equitable (accessible) digital health implementation, which includes elements of: co-design of tools with input from diverse communities during planning and development, selection of technologies that are inclusive, affordable and accessible, adapting tools to local needs and offering alternatives (possibly non-digital), and monitoring usage and outcomes across demographics to ensure equity.^100^

For the design of education apps for people with CRD, it has been suggested that the following aspects lead to the best outcomes for patients: using the principles of quality educational design, ensuring interactive experiences, patient co-design, and interaction with experts in the field of technology-enabled learning.^101^ Similarly, Murray (2022) identified design features that encouraged engagement by people with low digital health literacy, which included use of graphics, video, and audio to enhance accessibility, using relatable personas, reducing the reading age of content, and making key content available in different languages.^48^

### 5.3. Skills (Health services & voluntary/Community organisations)

Overall, digital confidence/self-efficacy is low in CRD patient groups and so it has been suggested that approaches to encourage online engagement are needed for people with CRD to build the confidence required to fully adopt DHTs into the self-management of their condition.^102^ Pimenta et al. (2023) overviewed research which has explored the facilitators which promote the adoption of digital health interventions in people living with CRD. They highlighted the importance of avoiding the assumption that all patients are ready for a digitally enabled treatment plan and the risk of a one-size-fits-all approach. Instead, they recommend that a personalised approach should be considered, to assess individual patient circumstances, including physical and mental health status, digital health literacy, self-efficacy, and home internet connectivity, to determine readiness for adopting a digital health technology pathway.^28^

In terms of the development of digital health literacy and skills, some practical actionable steps include: community-based digital health literacy initiatives such as training programmes in libraries, clinics, and community centres, training of staff to triage patients into digital or in-person pathways with shared decision making, and provision of technical support to patients during onboarding of DHTs.

### 5.4. Beliefs & trust (Researchers, innovators & healthcare management)

Motivation of HCPs and patients to engage in DHTs can be fostered via a number of factors, including: belief that the intervention/delivery is effective, a feeling that the DHT meets their needs and relates to them, the tool is presented in a simple and relatable way to boost confidence in using it, trust in the solution in terms of data privacy and usage, and the tool is promoted by community champions. It is imperative that the data collection and data sharing is in alignment with the needs and wishes of patients, communities and statutory bodies.^49^

### 5.5. Leadership & partnerships (Cross-sector organisations)

**Figure 1.**
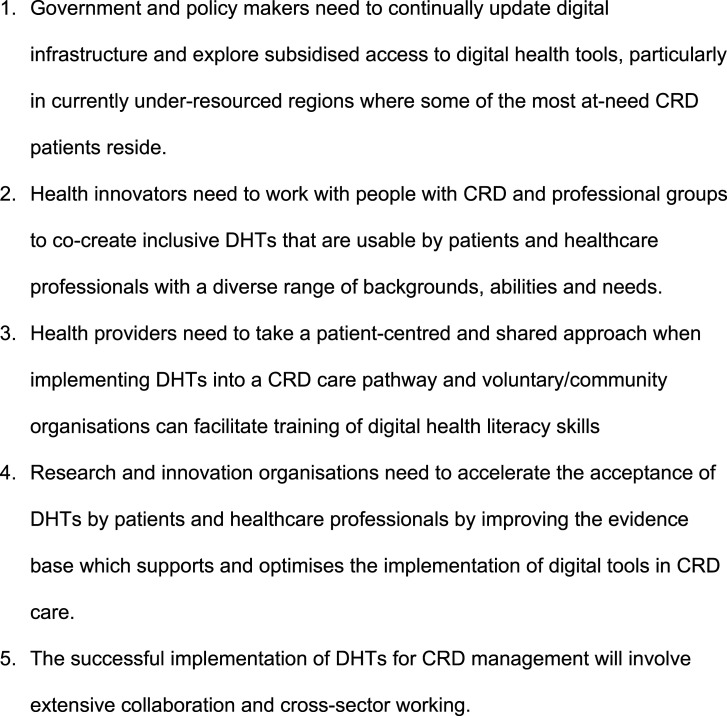
Summary of key recommendations.

The preceding considerations have highlighted that significant improvements to digital inclusion in the CRD patient population will need to be borne from multiple organisations working together. This involves large healthcare organisations, like the NHS in the UK, working with national and local government (for example on access to infrastructure), with VCSE organisations who can provide support to patients and digital health literacy training, with technology providers to promote the co-creation of accessible DHTs, with clinical staff to ensure digital pathways in CRD disease management are at least as effective as current practice, and with patient groups to ensure DHTs are deployed in manners which reduce the risk of digital exclusion among the most at-risk groups (Figure 1).

## 6. Conclusion

Given the health challenges typically faced by people living with CRD -mobility/activity limitations, anxiety/depression, social isolation - they are a patient group which simultaneously could derive huge benefit from adopting digital health technologies while at the same time being at a higher risk for digital exclusion. Establishing the extent to which digital exclusion negatively impacts on the healthcare experiences of people with CRD is made more difficult because typically digital exclusion is not a well-measured concept and the evaluation of DHTs often occurs without the involvement of underserved communities. There is a need to move beyond measuring inclusion as the ability to access, to measuring inclusion as the ability to benefit from DHTs performing specific tasks at times of most need. Strategies for ensuring traditionally underserved communities are included in evaluating digital health initiatives for CRD care should follow the principles of co-design. Elements should include: diverse participation in design, fair reimbursement for involvement, designing “with not for”, inclusion by default, evaluating burden as well as benefit, and diverse participation in evaluation. The barriers that lead to digital exclusion intersect at many different levels and include socioeconomic, demographic factors (age, ethnicity, education), geographical, digital literacy, design/accessibility and psychosocial factors. Solutions to mitigate digital exclusion in people with CRD need to operate at multiple scales with cross-sectoral collaboration and range from ensuring access to digital tools via large national infrastructure developments to ensuring digital health technologies are designed inclusively and frontline healthcare staff are trained to help patients engage with the tools.

We have reached a juncture where the presence of digital health interventions for the management of CRD is arguably a double-edged sword in terms of access and exclusion, where the deployment of DHTs may in fact exaggerate health inequities.^77,97,103,104^ Nevertheless, DHTs are likely to remain an important component of clinical care in people with CRD and therefore it is important to identify barriers to their use, particularly those likely to increase digital exclusion, and consider how best to overcome these.

## Data Availability

This article is a narrative review and does not involve the generation or analysis of primary data. All data supporting the findings of this review are available within the cited literature and can be accessed through the references provided.*
